# Contribution of a Circulating 2'-O-methylated MicroRNA Panel to the Diagnosis of Pancreatic Ductal Adenocarcinoma

**DOI:** 10.7150/jca.91716

**Published:** 2024-01-21

**Authors:** Zilin Yang, Jia Huang, Xuejiao Wu, Yufen Zhou, Yuming Tang, Ying Zhu, Baiwen Li, Xi Chen, Weiyan Yao

**Affiliations:** 1 Department of Gastroenterology, Ruijin Hospital, Shanghai Jiao Tong University School of Medicine, Shanghai, 200025, China.; 2 Department of Gastroenterology, Ruijin Hospital North, Shanghai Jiao Tong University School of Medicine, Shanghai, 201801, China.; 3 Department of Gastroenterology, Shanghai General Hospital, Shanghai Jiao Tong University School of Medicine, Shanghai, 201620, China.; 4 State Key Laboratory of Pharmaceutical Biotechnology, Jiangsu Engineering Research Center for MicroRNA Biology and Biotechnology, NJU Advanced Institute for Life Sciences (NAILS), School of Life Sciences, Nanjing University, Nanjing, Jiangsu, 210023, China.

**Keywords:** 2'-O-methylation, Circulating microRNA, Diagnosis, Pancreatic ductal adenocarcinoma, Plasma biomarkers

## Abstract

**Background:** We conducted an assessment of 2'-O-methylated (2'OMe) microRNAs (miRNAs) present in the circulation of individuals suffering from pancreatic ductal adenocarcinoma (PDAC). Subsequently, we devised a set of circulating 2'OMe miRNAs that can be utilized for the screening of PDAC patients within a group at increased risk.

**Methods:** A four-step, multicenter research was carried out. The initial screening phase involved analyzing 10 samples from patients with pancreatic ductal adenocarcinoma (PDAC) and 10 specimens from donors who were in good health. RNA sequencing was performed on these specimens after pre-treatment via NaIO4. The instruction and confirmation phases involved the use of 2'OMe miRNA profiling and multivariate analysis to examine applicant 2'OMe miRNAs in a sample of 248 individuals. In a prospective registration population of 135 individuals, a clinical screening panel was created and confirmed. The performance of individual 2'OMe miRNAs or the biomarker panel was evaluated using the receiver operating characteristic curve.

**Results:** Abnormal circulating 2'OMe miRNAs were detected in individuals suspected of pancreatic ductal adenocarcinoma (PDAC). A circulating panel of 3-2'OMe miRNAs, namely miR-28-3p, miR-143-3p, and miR-151a-3p, was subsequently identified. These miRNAs continually exhibited up-regulation in plasma samples of patients with pancreatic ductal adenocarcinoma (PDAC). The panel's area under the curve (AUC) was 1.0 in the experimental group and 0.928 in the verification cohort when comparing PDAC patients in all clinical stages to normal controls. During the application study, both the specificity and sensitivity were found to be 75.00% and 89.72% respectively, with an AUC value of 0.868. In the comparison between early-stage (I-II) PDAC patients and control subjects, the panel demonstrated an AUC of 1.0 in the training cohort and 0.924 in the validation population. In the application group the AUC was 0.810 (95% CI 0.729-0.876) in comparison to the high-risk symptomatic group.

**Conclusion:** Abnormal circulating 2'OMe miRNAs were detected in individuals with pancreatic ductal adenocarcinoma (PDAC). Consequently, we devised a 2'OMe miRNA biological marker panel that holds promise as an initial detection tool for PDAC.

## Background

Pancreatic ductal adenocarcinoma (PDAC) is considered the third most significant contributor to cancer mortality, mostly because to its very poor 5-year survival rate of about 10% in the United States 1. The absence of timely detection has impeded the implementation of efficacious therapy aimed at decelerating the advancement of PDAC 2, 3**.** The presently accessible markers for PDAC, such as cancer antigen 19-9 (CA19-9) and carcinoembryonic antigen (CEA), exhibit limited sensitivity for the purpose of PDAC screening, particularly during the first phases. There exists a prevailing need for the development of targeted screening tests for PDAC that has yet to be adequately addressed 4-7.

MicroRNAs (miRNAs) are a class of short noncoding RNA molecules, typically consisting of 18-22 nucleotides in length 8, 9. These molecules play a crucial role in regulating several physiological and pathological processes as endogenous regulators, especially at the post-transcriptional level. Furthermore, there has been significant research conducted on miRNAs to investigate their possible impact on the control of tumor immunity by directly targeting genes associated with immune activation or repression 10. The process of tumorigenesis is a complex and protracted phenomenon that occurs under the regulatory control of the immune system 11. Circulating microRNAs (miRNAs), which possess greater accessibility compared to miRNAs found in tissues, have the potential to serve as noninvasive biomarkers for several conditions, including PDAC 12. The molecular stability in the extracellular environment is significantly impacted by several modifications of miRNAs, including methylation, uridylation, and adenylation. Among these options, methylation has garnered growing interest. New research has provided verification that some miRNAs include methyl markings that have the potential to modify stability and target recognition. These methyl marks include 5-methylcytosine (5mC), N6-methyladenosine (m6A), 3-methylcytosine, and N1-methyladenine (m1A) 13. In recent studies, it has been shown that the incorporation of 3'-terminal 2'-O-methylated (2'OMe) alteration might enhance the stability of miRNA molecules. Notably, the presence of 3'-terminal 2'OMe miRNAs in humans was first seen in non-small cell cancer (NSCLC) tissue 14, 15.

The current investigation assessed the existence of circulating 2'OMe miRNAs in individuals diagnosed with PDAC and then confirmed the results in several cohorts. Subsequently, we proceeded to fabricate a plasma panel consisting of 2'-O-methylated miRNAs, with the aim of establishing a noninvasive biomarker for the identification of PDAC. Furthermore, we conducted an assessment of its efficacy in a prospective cohort.

## Material and methods

### Study design and patient cohort

In a four-stage, multicenter investigation, we identified and validated the profile of circulating 2'OMe miRNA biomarkers for PDAC in a total of 403 individuals. During the screening stage, we assessed the levels of 521 circulating miRNAs in a group consisting of 10 patients with PDAC and 10 control individuals who were assigned accordingly. In the training stage, which made of 10 PDAC patients and 10 matched healthy controls, we recognized applicant 2'OMe miRNAs and multi-2'OMe miRNA panel. In the validation stage, a cohort of 105 people with PDAC as well as a control group consisting of 103 healthy donors of blood and 20 individuals with chronic pancreatitis (CP) were chosen to further verify the diagnostic value of multi-2'OMe miRNA panel. In these three stages, all patients were recruited in Ruijin Hospital and the General Hospital of Eastern Theater Command between 2019 and 2021. The patient with PDAC were newly diagnosed PDAC in Ruijin Hosipital. Blood was collected prior to any treatment. Controls were matched by sex, age at sampling, and sampling date (± 3 months). The non-tumor control group with CP were diagnosed by imaging, laboratory indicators and clinical manifestations in Ruijin Hospital. Matched normal control subjects were recruited from healthy physical examination population. Among them, we devoloped exclusion criteria to decrease the influence of other physical or pathological variables on plasma 2'OMe miRNAs production, including age < 18 years old, age > 80 years old, history of chemoradiotherapy, history of complete or partial removal of the pancreas, strict immune diseases, severe organ failure (liver failure, heart failure, or kidney failure), other tumors except PDAC, poor sample quality, and lack of complete clinical information. In addition to the aforementioned cohorts, a prospective multicenter step with 193 participants with abdominal discomfort and elevated CA19-9 expression (higher than 37 U/mL) confirmed the multi-2'OMe miRNA panel, including 125 from Center 1 (Shanghai Ruijin Hospital), 37 from Center 2 (Shanghai Ruijin Hospital North), 36 from Center 3 (Shanghai General Hospital South) between 2019 and 2021. The abdominal discomfort includes abdominal pain, dyspepsia, jaundice, nausea, vomitt and weight loss. According to the above inclusion and exclusion criteria, a total of 135 participants were finally recruited, including 91 patients at Center 1 (Shanghai Ruijin Hospital), 24 patients at Center 2 (Shanghai Ruijin Hospital North), and 20 patients at Center 3 (Shanghai General Hospital South). PDAC was identified through pathological assessments in this stage. 16 The study adhered to the STARD regulations for carrying out and disclosing diagnostic accuracy studies.

Patient medical information, such as sex, age, disease history, CA19-9 level, tumor location, and clinical stage, were gathered. The studies received approval and consent from the Ruijin Hospital Ethics Committee, with the reference number 2019-158. 193 participants enrolled for study collected from 3 hospitals (125 from Shanghai Ruijin Hospital, 37 from Ruijin Hospital North, 36 from Shanghai General Hospital South).

### Extraction of Plasma RNA

Total RNA extraction for Illumina sequencing by synthesis (SBS) technology was performed using TRIzol@ls Reagent (Invitrogen, USA). According to the instructions for the qRT-PCR experiment, we extracted miRNA from a 200-L plasma sample using the miRNeasy Serum/Plasma Kit (Qiagen, Germany). The whole RNA was kept at 80 °C in a solution of diethylpyrocarbonate (DEPC) water.

### NaIO_4_ oxidation and small RNA sequencing in the screening step

RNA molecules containing a 2ʹ-O-methyl (2ʹOMe) modification at the 3ʹ-terminal are capable of withstanding the powerful oxidation effects induced by NaIO4. Consequently, we validated 2'OMe miRNAs by oxidizing circulating short RNA isolated from PDAC and plasma of healthy donors using NaIO4 and Illumina SBS technology. The samples were split into two groups, with the group that had not been subjected to oxidation serving as a control. This group had not been pretreated with NaIO4. Instead, NaIO4 was used on the oxidation group to eliminate any short RNA that lacked a 3'-terminal 2'-O-methyl group. The oxidation groups were treated with a solution containing 400 ng of small RNA and 80 mM of NaIO4 dissolved in 5× borate buffer (150 mM borax and 150 mM boric acid at pH 8.6), which was freshly prepared from Sigma Aldrich, USA. To investigate the non-oxidation groups, a set of reactions was prepared by substituting a 5× borate buffer for NaIO4. The solution was incubated at room temperature for 30 minutes and then subjected to ethanol precipitation. The RNA Clean & Concentrator-5 Kit, manufactured by Zymo in the United States, was employed for the purification of small RNAs. The RNAs obtained were dissolved in DEPC water prior to sequencing.

17 We employed Illumina SBS technology to analyze circulating miRNAs. After performing polyacrylamide gel electrophoresis (PAGE) purification on RNA molecules smaller than 30 base pairs (bp), adapters were ligated to both the 5' and 3' ends of the RNA. Adapter primers were employed for 17 cycles to amplify small RNA molecules, while PAGE gels were utilized to separate fragments of approximately 90 bp. The manufacturer's instructions were followed to obtain pure DNA for cluster creation and sequencing analysis. An Agilent 2100 bioanalyzer, specifically the Agilent DNA 1000 Reagents, was used for this purpose. The image files from the sequencer were processed to obtain data of high digital quality. The subsequent steps included data acquisition, evaluation of sequence quality and depth, analysis of small RNA length distribution, and filtering of contaminated reads. The Smith-Waterman algorithm was employed to align unambiguous reads with the miRBase database 21.0 following the removal of adaptor sequences. The total frequency of sequencing for each sample was then normalized to 1,000,000.

### qRT-PCR for stem-loop primer method and tailing system in the training, validation, and application steps

In our study, we employed two qRT-PCR systems, specifically a stem-loop primer and a poly (A)-tailed method, which have been already described 18. The standard curve was utilized to provide absolute measurement of the circulating miRNAs that were tested. The current study lacks an internal reference due to the absence of an accord regarding the optimal internal reference for measuring circulating miRNAs.

LightCycler®480 (Roche, USA), TaqMan miRNA probes, and the miScript Primer Assay (Qiagen) were used in conjunction with the stem-loop primer and poly (A)-tailed RT-qPCR studies as recommended by the manufacturer. The reactions were triply examined.

### Synthetic miRNA oligonucleotides

Both unmethylated and 2'-O-methylated miRNA oligonucleotides were synthesized at a lab (GenePharma, China). Stem loop primer quantitative real-time polymerase chain reaction calibration required a reaction concentration of 0.1 to 1.0 M. The study's oligonucleotide sequences are provided in Table S1.

### Statistical analysis

Statistical analysis was conducted using GraphPad Prism 7 (GraphPad Software, USA), MedCalc (MedCalc Software Ltd, Belgium), and SPSS 26.0 (IBM, USA). The mean ± SEM was presented using data obtained from a minimum of three independent experiments. The statistical comparison between two groups was conducted using Student's t-test. Statistical significance was defined as p-values less than 0.05.The panel generated a numerical PDAC risk score for each subjects using SPSS. Using the circulating multi-2'OMe miRNA panel, a cancer prediction score was generated based on the most optimal sensitivity and specificity combination. The risk score was calculated using a linear regression model of the measured expression levels of the circulating 2'OMe miRNAs in the panel. The selected probability of 0.50 or more was defined as a positive test result. The diagnostic value of individual 2'OMe miRNAs or the multivariate biomarker panel in PDAC was assessed using the receiver operating characteristic curve (ROC) and the area under the curve (AUC) was used as the performance indicator.

## Results

### Identification of PDAC-associated circulating 2'OMe miRNAs

Ten PDAC patients and ten age- and sex-matched controls were used as a screening cohort, and the resulting list of circulating 2'OMe miRNAs was further validated in a second cohort of ten PDAC patients and ten controls. Table 1 presents the clinicopathological characteristics of both the screening and training cohorts.

**Figure 1:**
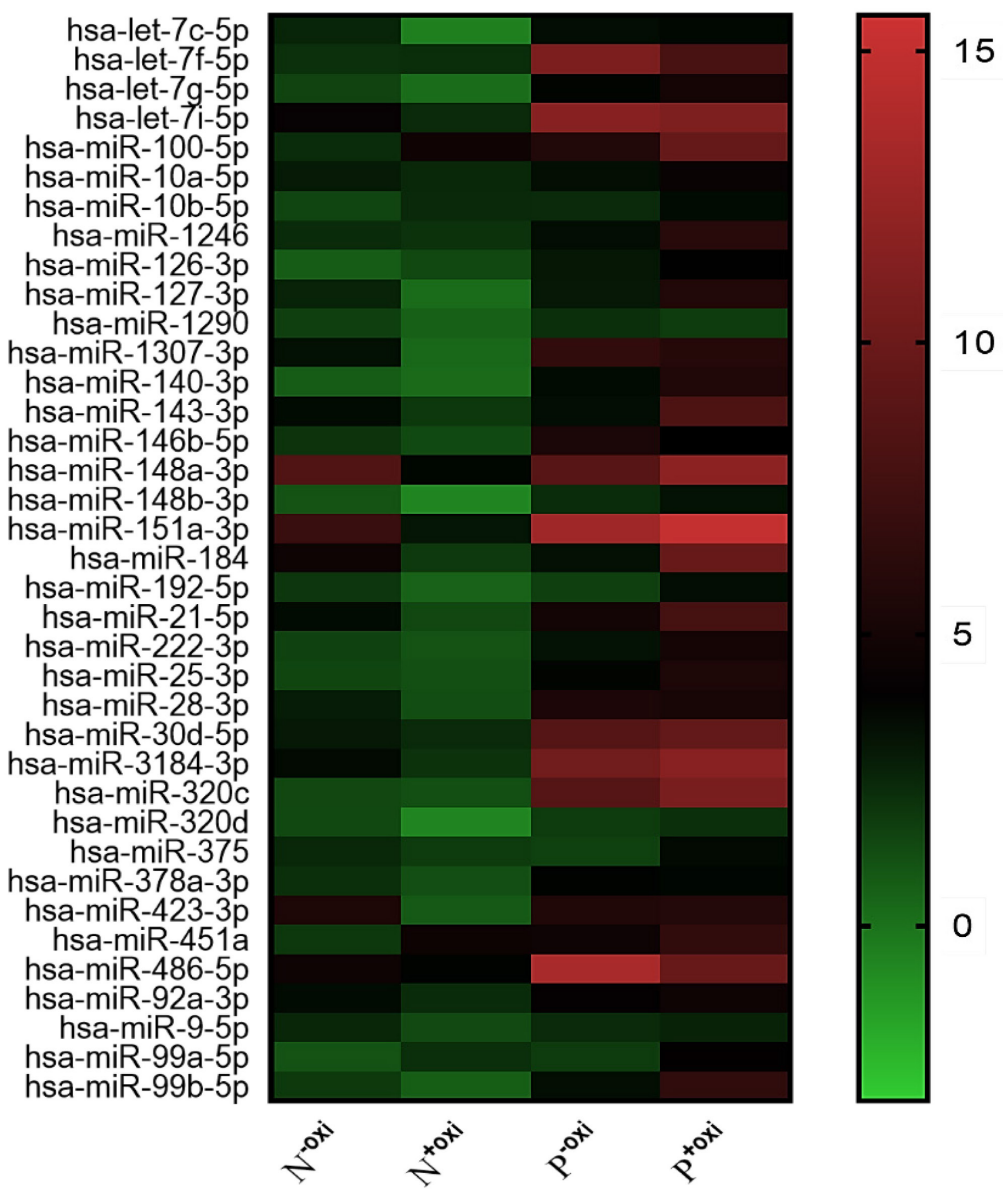
A methylation status heatmap of circulating miRNAs, divided into four categories based on sequencing data. The N^-oxi^ NC group, untreated with oxygen. Oxidation pre-treatment for the N^+oxi^ NC group. P^-oxi^ PDAC category without pre-treatment with oxidation. Group P^+oxi^ PDAC underwent pretreatment with oxidation.

Following pre-treatment with oxidation, 521 circulating miRNAs were analyzed using Illumina SBS technology for the screening population. Out of the 521 measured circulating miRNAs, 68 miRNAs were identified in the groups that did not undergo oxidation pretreatment procedures, with an expression level of at least 100 copies. In all, 37 potential 2'-O-methylated (2'OMe) miRNAs were identified, and a heatmap comparing circulating miRNAs with and without methylation in PDACs and NCs revealed a striking difference (Figure 1). The PDAC group consisted of patients diagnosed with early-stage pancreatic ductal adenocarcinoma (40% stage I and 30% stage II).

**Figure 2:**
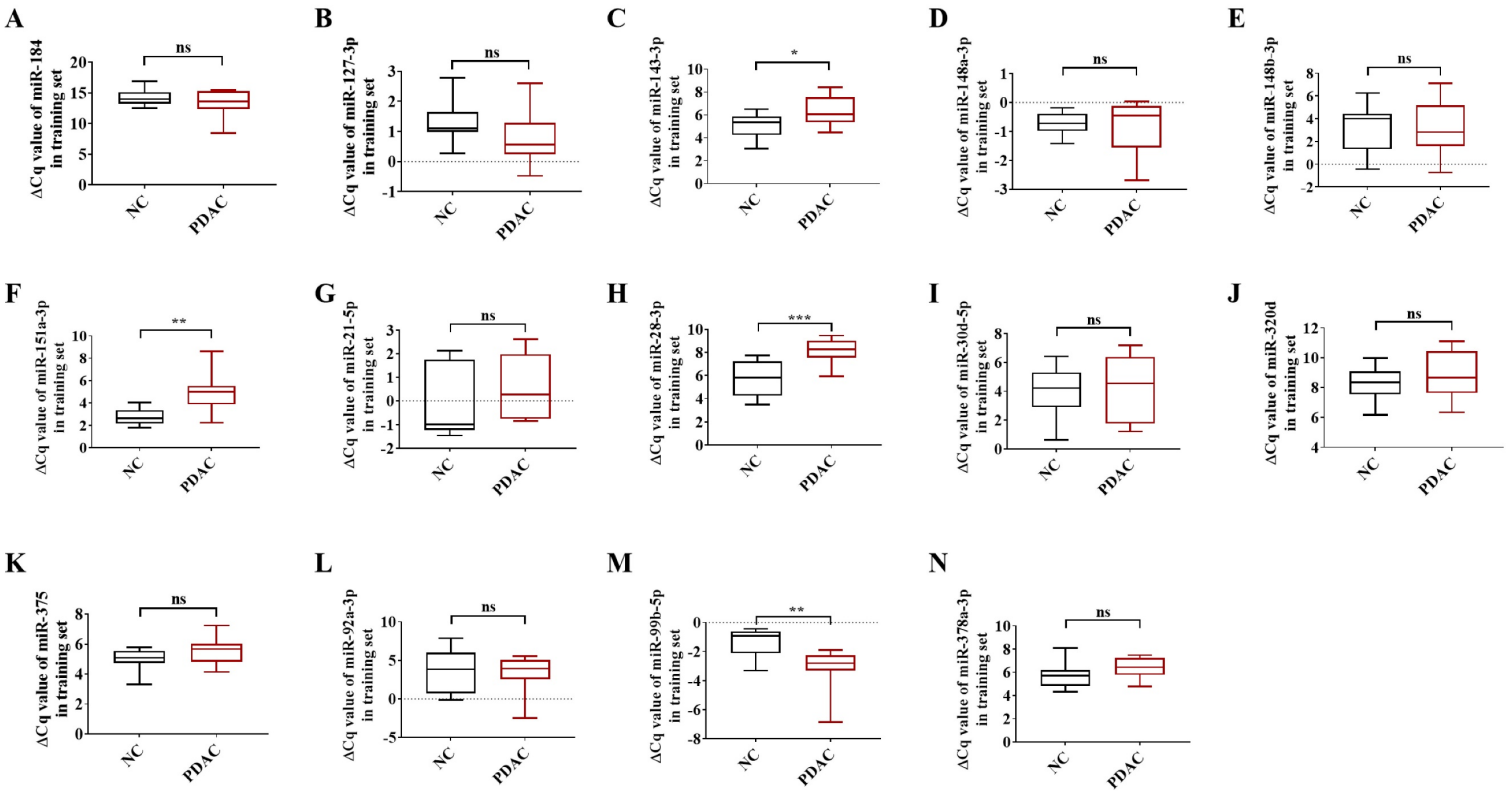
In the training step, the ΔCt value of 14 candidate 2'OMe miRNAs was determined using qRT-PCR with stem-loop primer and tailing system. The following microRNAs were analyzed: miR-184 (A), miR-127-3p (B), miR-143-3p (C), miR-148a-3p (D), miR-148b-3p (E), miR-151a-3p (F), miR-21-5p (G), miR-28-3p (H), miR-30d-5p (I), miR-320d (J), miR-375 (K), miR-92a-3p (L), miR-99b-5p (M), and miR-378a-3p (N). The symbols used to denote statistical significance are as follows: P < 0.05 indicates significance, **P < 0.01 indicates high significance, ***P < 0.001 indicates very high significance, and "ns" indicates no significant difference.

To be deemed significantly up-regulated, a candidate miRNA with 2'OMe alteration needed to have sequencing reads that matched the following criteria: > 0 copies in all groups, > 100 copies in non-oxidation groups, fold change (FC) PDAC 0.5, and FC NC 0.5. Out of the 37 identified circulating 2'OMe miRNAs, nine were found in both the PDAC and NC groups with no variation in copy numbers. Among the circulating 2'OMe miRNAs, 26 were up-regulated in the PDAC group, while two were down-regulated (Table S2). We selected 14 out of the top 20 2'OMe miRNAs (miR-127-3p, miR-143-3p, miR-148a-3p, miR-148b-3p, miR-151a-3p, miR-184, miR-21-5p, miR-28-3p, miR-30d-5p, miR-320d, miR-375, miR-92a-3p, miR-99b-5p, miR-378a-3p) for further verification. We used the value of FC (P+oxi/P-oxi) / FC (N+oxi/N-oxi) as a reference for this selection. In addition, we identified 14 potential 2'OMe miRNAs in the training set, which consisted of 10 cases of pancreatic ductal adenocarcinoma (PDAC) and 10 normal controls (NCs). This was accomplished using two qRT-PCR systems: the stem loop primer and tailing method. Three 2'OMe miRNAs, specifically miR-28-3p, miR-143-3p, and miR-151a-3p, exhibited a notable amplification delay (Figure 2), thereby confirming the presence of the 2'OMe modification in circulating miRNAs.

Subsequently, we performed dilutions of synthetic miRNAs at concentrations ranging from 10^-2^μM to 10^-8^ μM. We then assessed the extent of amplification using two distinct RT-qPCR methods. In addition to synthetic non-2'OMe miRNAs, the poly (A) tail qRT-PCR system demonstrated a noticeable lag in amplification over synthetic 2'OMe miR-28-3p, 2'OMe miR-143-3p, and 2'OMe miR-151a-3p at equivalent RNA concentrations (Figure S1). This finding provides additional proof supporting the effectiveness of this technique for detecting the 2'OMe modification. In addition, we conducted an analysis of the cycle threshold (Ct) admire of different miRNAs so as to assess the methylation ratio. Our findings revealed a stronger correlation between the Ct value of poly (A)-tailed qRT-PCR and miRNA methylation, which followed a second-order polynomial curve (Figure S2). The nonlinear regression tackle was used to evaluate the methylation percentage of each circulating small RNA in a sample of biological matter. In the training set, the methylation ratios of circulating miR-28-3p, miR-143-3p, and miR-151a-3p in PDAC were 1.03, 0.56, and 0.26, respectively, as determined by standard curves. These proportions were found to be greater than those observed in the NC groups, respectively (Figure S3).

**Figure 3:**
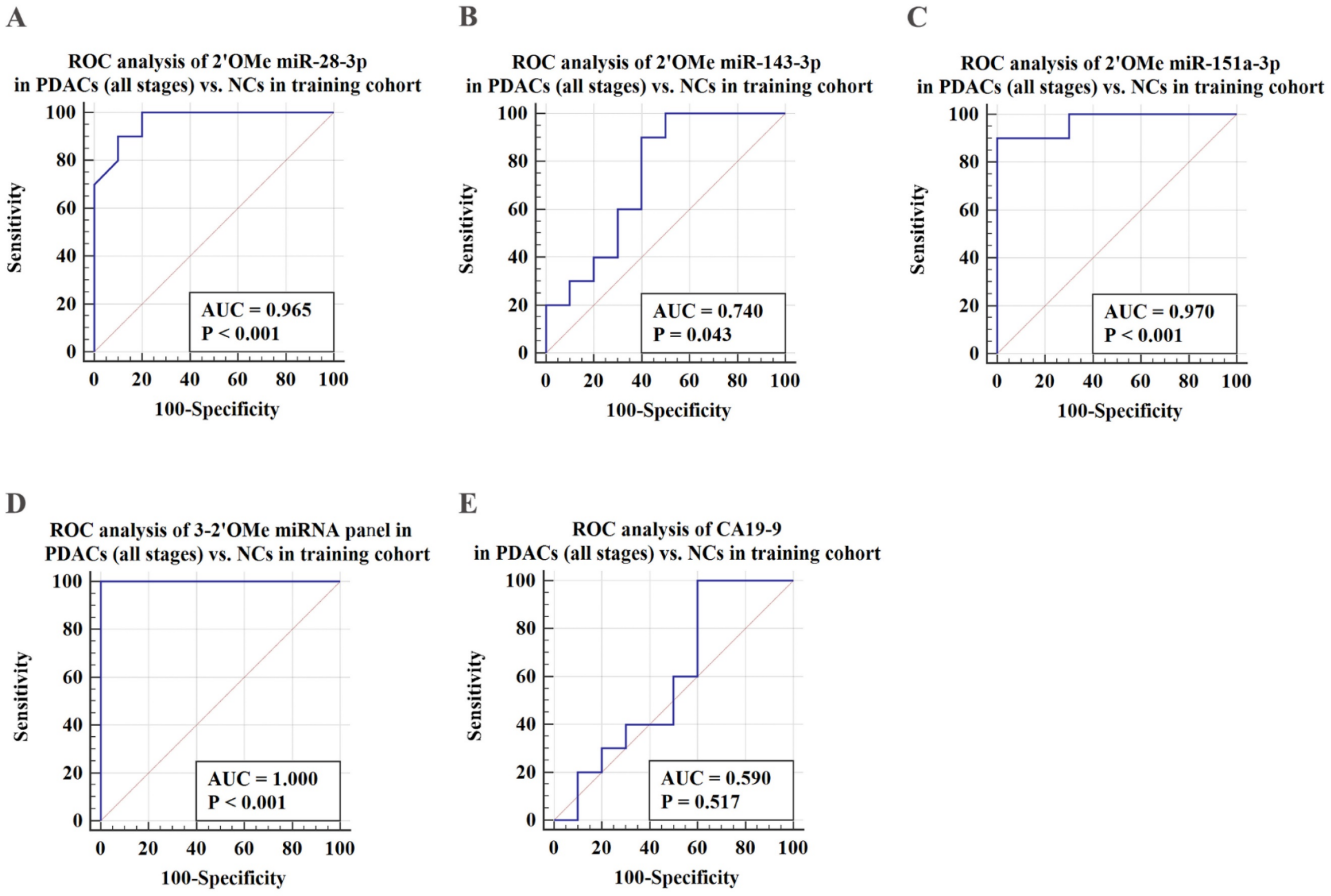
Validation of PDAC-associated 2'OMe miRNA biomarkers and the accuracy of detecting a multi-miRNA biomarker panel in the training set. ROC curves were generated for the following biomarkers in the detection of pancreatic ductal adenocarcinoma (PDAC) patients across all clinical stages: 2'OMe miR-28-3p (A), 2'OMe miR-143-3p (B), 2'OMe miR-151a-3p (C), a multi-miRNA biomarker panel (D), and CA19-9 (E). AUC was employed to assess the accuracy of PDAC detection.

ROC analysis was conducted to assess the capacity to differentiate PDAC cases from identical controls using indicative circulating 2'OMe miRNAs. Results demonstrated that individual 2'OMe miRNAs (2'OMe miR-28-3p, 2'OMe miR-143-3p, and 2'OMe miR-151a-3p) had AUC values of 0.965 (95% CI 0.774 to 1.0), 0.740 (95% CI 0.498 - 0.907), and 0.970 (95% CI 0.781 - 1.0), respectively (Figure 3B-D). Furthermore, the combination of these three 2'OMe miRNA biomarkers (2'OMe miR-28-3p, 2'OMe miR-143-3p, and 2'OMe miR-151a-3p) yielded an AUC of 1.000 (95% CI 0.832-1.0), outperforming CA19-9 with an AUC of 0.590 (Figure 3E-F).

### Verification of the 3-2'OMe miRNA panel

**Figure 4:**
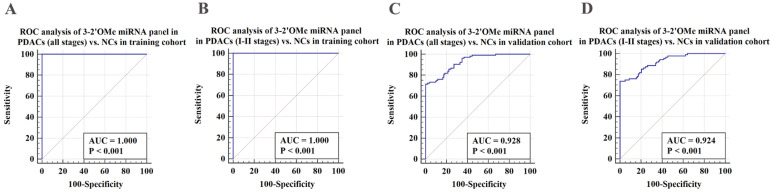
Validation of the PDAC 3-2'OMe miRNA panel detection in separate groups. ROC curve analyses were conducted to evaluate the performance of the 3-2'OMe miRNA panel in detecting pancreatic ductal adenocarcinomas (PDACs) in both all-stage and early-stage cases. The analyses were carried out separately for the training and validation cohorts.

Subsequently, we assessed the efficacy of the 3-2'OMe miRNA panel, which was discovered during the course of the group, in the validation group (Table 2). In the validation set, the methylation ratios of circulating miR-28-3p, miR-143-3p, and miR-151a-3p in PDAC were 1.01, 0.56, and 0.26, respectively, in CP were 1.03 determined by standard curves. These proportions were found to be greater than those observed in the NC group, but little difference in the CP groups (Figure S4). In the meantime, a similar pattern of performance was seen for the 3-2'OMe miRNA panel in the validation sample. The panel distinguished PDAC cases from matched controls with a high AUC of 0.928 (95% CI: 0.884 - 0.959) in the validation cohort, similar to the training set (Figure 4A, 4C). When comparing early-stage PDAC (stages I-II) to the matched controls, the AUC was 0.924 (95% CI: 0.877 - 0.957) (Figure 4D), whereas the AUC was 1.000 in the training set (Figure 4B). These results show that the panel is highly accurate at distinguishing PDAC cases from matched controls. The findings suggest that the three-2'OMe miRNA panel (2'OMe miR-28-3p, 2'OMe miR-143-3p, and 2'OMe miR-151a-3p) has the potential to be a valuable diagnostic tool for the early identification of PDAC. We utilized the panel for prospective validation.

### Application of the 3-2'OMe miRNA panel

#### Study population

**Figure 5:**
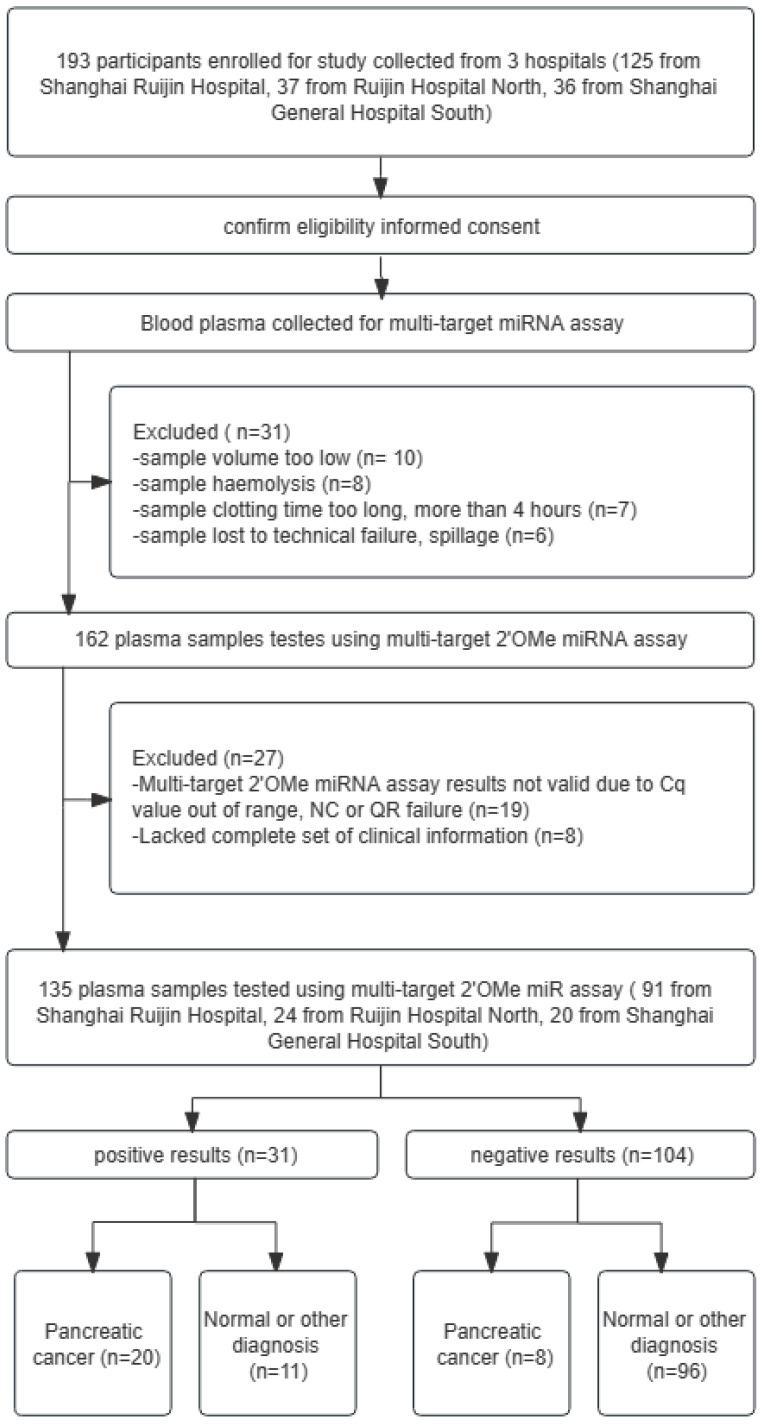
A flowchart illustrating the design of a prospective validation study, in accordance with the guidelines set forth by the Standards for Reporting of Diagnostic Accuracy Studies. QR codes are a type of barcode that can be scanned using a smartphone or other scanning device to quickly access information or resources.

Patients complaining of stomach pain and having elevated levels of CA19-9 were studied using the 3-2'OMe miRNA panel (Figure 5). Table 3 displays the clinicopathological features that were observed. A cohort of 193 individuals underwent CA19-9 testing, and plasma samples were collected for the assessment of the 3-2'OMe miRNA panel. In all, 31 participants couldn't be included in the miRNA study because of poor quality of the specimens. Only 27 samples out of the remaining 162 were found to have accurate findings. The remaining samples did not provide reliable data owing to inadequate clinical information or incorrect expression ranges. A total of 135 participants had detectable results for both the 3-2'OMe-miRNA assay and CA19-9 tests. A total of 28 cases of pancreatic ductal adenocarcinoma (PDAC) have been identified through surgical pathology. These cases were observed in individuals who presented with abdominal symptoms and had elevated levels of CA19-9, with a prevalence rate of 20.74%.

#### Assay performance

**Figure 6:**
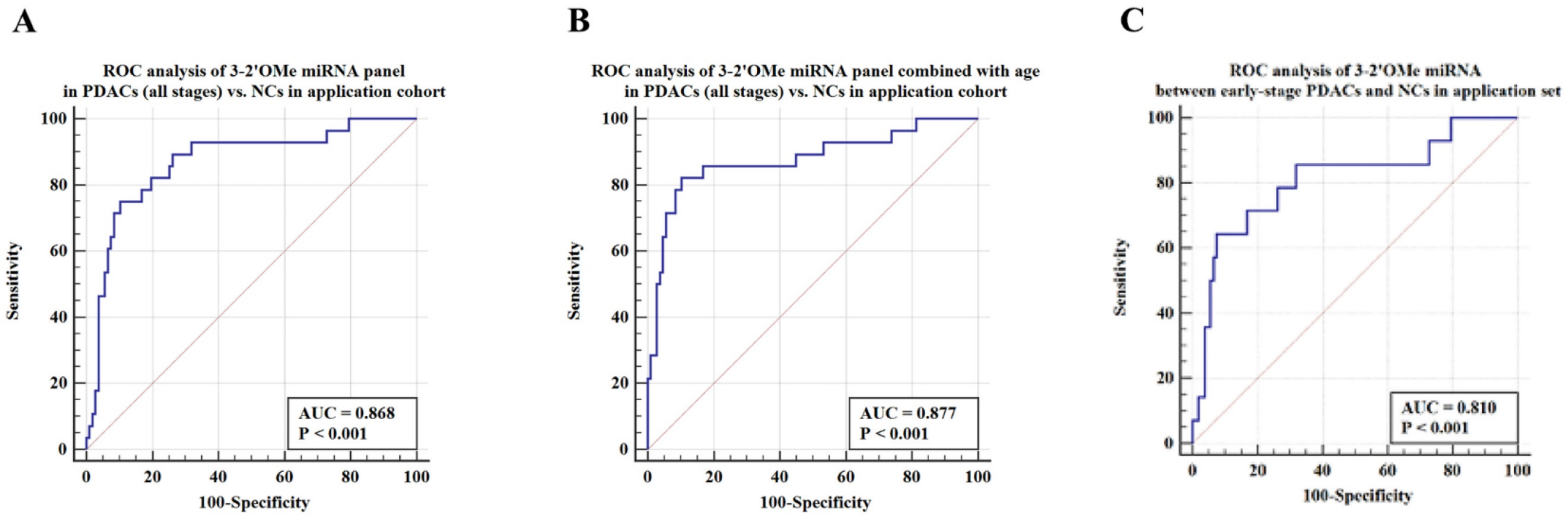
Validation of the accuracy of a multi-miRNA biomarker panel associated with pancreatic ductal adenocarcinoma (PDAC) in a specific application dataset. This study conducted three ROC analyses in an application cohort. The first analysis examined the 3-2'OMe miRNA panel in PDACs (all stages) compared to NCs. The second analysis combined the 3-2'OMe miRNA panel with age in PDACs (all stages) compared to NCs. The third analysis focused on the 3-2'OMe miRNA panel in early-stage PDACs compared to NCs.

Among this sample group, the AUC for the panel was 0.868 (Figure 6A). When patient age is taken into account, the sensitivity of the panel of 3-2'OMe miRNA increases to 82.14% and the specificity increases to 89.72% (Figure 6B), with an AUC of 0.877. In the comparison between early-stage PDAC (stages I-II) and the high-risk symptomatic group, the area under the curve (AUC) was found to be 0.810 (95% CI: 0.729-0.876) (Figure 6C).

The panel of 3-2'OMe miRNA detected 20 out of 28 cases of pancreatic ductal adenocarcinoma (PDAC), resulting in a sensitivity of 75.00% (95% CI: 55.1%-89.3%) and a specificity of 89.72% (95% CI: 82.3%-94.8%), as illustrated in Figure 6a. The PPV was 64.52% and the NPV was 92.31%.

## Discussion

19, 20 Because of their amazing stability and noninvasive sampling techniques, circulating miRNAs have attracted a lot of interest in solid tumors lately. Circulating miRNAs are being proposed as possible markers for pancreatic ductal adenocarcinoma (PDAC) identification.

21, 22 More and more research suggests that miRNA methylation may be a significant sort of alteration that contributes to both self-stability and functional variability. Fifteen miRNAs with 3' terminal 2'OMe alteration have been identified in plants and Drosophila. ^15^This modification first appeared in mammals in the year 2020 15. In addition, it has been observed that miR-21-5p with a 3'-terminal 2'-OMe modification exhibits a notably extended half-life compared to unmethylated miR-21-5p in lung tissues. The scarcity of data on circulating 2'OMe miRNAs is attributed to the challenges in detecting these small RNAs. The identification process involves the use of pre-treatment NaIO4 oxidation in conjunction with either northern blot or mass spectrometry, which necessitates a substantial quantity of samples. 18 A new investigation introduced a poly (A) tail qRT-PCR technique that enables the detection of 2'OMe miRNAs using a reduced amount of sample material. In this study, we employed NaIO4 oxidation treatment along with Illumina SBS technology and poly (A) tail qRT-PCR to identify circulating 2'OMe miRNAs in individuals with pancreatic ductal adenocarcinoma (PDAC) and in a group serving as a control of healthy people. The present research was the initial to discover the presence of circulating 2'OMe miRNAs in PDAC. 23 Previous studies have established that m6A methylation plays a role in modifying miRNAs, thereby regulating various processes such as immune response, tumorigenesis, and metastasis in pancreatic ductal adenocarcinoma (PDAC). Based on this knowledge, we hypothesized that the presence of abnormal 2'OMe miRNAs might be linked to an abnormal immune microenvironment in PDAC.

In order to assess the viability of using circulating 2'OMe miRNAs as biomarkers for cancer detection, we initially examined 28 potential circulating candidate 2'OMe miRNAs in both PDAC patients and individuals without the disease. Fourteen 2'OMe miRNAs were chosen as plasma candidates for testing. During the training and validation stages, it was observed that three specific 2'OMe miRNAs (miR-28-3p, miR-143-3p, and miR-151a-3p) consistently exhibited up-regulation in plasma samples of patients with pancreatic ductal adenocarcinoma (PDAC). A multivariate panel was developed to address the issue of low detection precision caused by tumor heterogeneity. The 3-2'OMe miRNA panel demonstrated superior performance in identifying patients with pancreatic ductal adenocarcinoma (PDAC) compared to CA19-9, as observed in both the training and validation sets. Furthermore, the 3-2'OMe miRNA panel demonstrated clinical significance in detecting early-stage patients with pancreatic ductal adenocarcinoma (PDAC). Our findings indicate that assessing the methylation of miRNA holds promise as a diagnostic approach.

The current diagnostic methods for pancreatic ductal adenocarcinoma (PDAC) involve the use of biopsy, imaging techniques, and tumor biomarker testing. However, CA19-9 is also raised in non-pancreatic disorders such as biliary tract disease and stomach pain, and has therefore been utilized primarily as a plasma marker for screening and monitoring recurrence of PDAC. We included 135 participants with elevated CA19-9 levels and abdominal symptoms in our prospective cohort study. The panel demonstrated a sensitivity of 75.00% and specificity of 89.72% in accurately differentiating PDAC cases from non-PDAC diseases in the high-risk groups. The area under the receiver operating characteristic curve (AUC) for the panel alone was 0.868. However, when age was included in the analysis, the AUC increased to 0.877. The data indicate that the panel may serve as a biomarker for the diagnosis of pancreatic ductal adenocarcinoma (PDAC). Nevertheless, the research had a few constraints. To begin, we did not investigate the functions of 2'OMe miRNAs in PDAC initiation, progression, and maintenance. Furthermore, it is necessary to assess the panel in a diverse population sample, including both individuals at elevated risk for pancreatic ductal adenocarcinoma (PDAC) and the general population as a whole.

## Conclusion

In summary, our research has successfully identified circulating 2'OMe miRNAs that are related with PDAC in patients. Additionally, a panel of multi-2'OMe miRNA biomarkers was designed with the aim of serving as a possible screening instrument for the detection of PDAC.

## Supplementary Material

Supplementary figures and tables.

## Figures and Tables

**Table 1:** Clinicopathological characteristics in screening and training cohort

	Screening cohort		Training cohort	
Variable	Case (%)	Controls (%)	Case (%)	Controls (%)
Number	10	10	10	10
Sex				
Male	6(60.00)	5(50.00)	6(60.00)	5(50.00)
Female	4(40.00)	5(50.00)	4(40.00)	5(50.00)
Age				
≤60	5(50.00)	5(50.00)	3(30.00)	4(40.00)
>60	5(50.00)	5(50.00)	7(70.00)	6(60.00)
TNM stage				
I	4(40.00)		4(40.00)	
II	3(30.00)		3(30.00)	
III	2(20.00)		2(20.00)	
IV	1(10.00)		1(10.00)	

**Table 2:** Validation cohort clinicopathological characteristics

	Validation cohort		
Variable	PDAC (%)	NC (%)	CP (%)
Number	105	103	20
Sex			
Male	64(60.95)	59(57.28)	11(55.00)
Female	41(39.05)	44(42.72)	9(45.00)
Age			
≤60	31(29.52)	37(35.92)	12(60.00)
>60	74(70.48)	66(64.08)	8(40.00)
TNM stage			
I	40(38.10)		
II	48(45.71)		
III	12(11.43)		
IV	5(4.76)		

**Table 3:** The clinicopathological characteristics of the application cohort

	Application cohort	
Variable	Cases (%)	Controls (%)
**Number**	28	107
**Sex**		
Male	21(75.00)	59(61.29)
Female	7(25.00)	48(38.70)
**Age**		
≤60	16(57.14)	41(36.56)
>60	12(42.86)	66(63.44)
**TNM stage**		
I	8(28.57)	
II	6(21.43)	
III	5(17.85)	
IV	9(32.14)	

## Abbreviations

PDAC: Pancreatic ductal adenocarcinoma
CA19-9: cancer antigen 19-9
CEA: carcinoembryonic antigen
miRNAs: MicroRNAs
5mC: 5-methylcytosine
m6A: N6-methyladenosine
m1A: N1-methyladenine
2'OMe: 2'-O-methylated
NSCLC: small cell lung carcinoma
CP: chronic pancreatitis
SBS: sequencing by synthesis
qRT-PCR: quantitative reverse-transcription polymerase chain reaction assays
DEPC: diethyl pyrocarbonate
PAGE: polyacrylamide gel electrophoresis
Bp: base pairs
ROC: receiver operating characteristic curve
AUC: area under the curve
NC: negative control
FC: fold change
Ct: cycle threshold
CI: confidence interval
PPV: positive predictive value
NPV: negative predictive value
