# Supplementary material for: Contribution of a Circulating 2'-O-methylated MicroRNA Panel to the Diagnosis of Pancreatic Ductal Adenocarcinoma
Source: J Cancer. 2024 Jan 21;15(6):1583–92. doi: 10.7150/jca.91716 (PMC10869982; doi:10.7150/jca.91716)
Supplement: Supplementary file 1 — Supplementary figures and tables. [file jcav15p1583s1.pdf]

Supplementary Material

Supplementary Tables:

Table S1: Synthetic miRNA oligonucleotides in this study

miRNA ID	Sequence (5'–3')
miR-28-3p	CACUAGAUUGUGAGCUCCUGGA
miR-28-3p-2'OMe	CACUAGAUUGUGAGCUCCUGG2'OMeA
miR-143-3p	UGAGAUGAAGCACUGUAGCUC
miR-143-3p-2'OMe	UGAGAUGAAGCACUGUAGCU2'OMeC
miR-151a-3p	CUAGACUGAAGCUCCUUGAGG
miR-151a-3p-2'OMe	CUAGACUGAAGCUCCUUGAG2'OMeG

Table S2: Differently expressed plasma 2'OMe miRNAs in PDAC pooled samples

(26 up-regulated 2'OMe miRNAs and 2 down-regulated 2'OMe miRNAs)

miRNA ID	**N^{-oxi}** (TPM)	**N^{+oxi}** (TPM)	**FC** (N^{+oxi} /N^{-oxi})	**P^{-oxi}** (TPM)	**P^{+oxi}** (TPM)	**FC** (P^{+oxi} /P^{-oxi})	**FC**(P^{+oxi}/P^{-oxi}) **/FC**(N^{+oxi}/N^{-oxi})
hsa-miR-184	9.24	5.46	0.07	7.82	12.16	20.23	289.00
hsa-miR-127-3p	6.74	2.36	0.05	7.36	9.89	5.75	115.00
hsa-miR-423-3p	9.76	3.53	0.01	9.86	9.94	1.06	106.00
hsa-miR-143-3p	8.13	5.5	0.16	8.02	11.32	9.85	61.56
hsa-let-7c-5p	6.72	0.77	0.02	8.04	8.34	1.22	61.00
hsa-miR-192-5p	5.66	2.96	0.15	5.1	8.05	7.7	51.33
hsa-miR-148b-3p	3.94	0.19	0.07	6.22	7.72	2.84	40.57
hsa-miR-21-5p	8.13	4.61	0.09	9.41	11.09	3.21	35.67
hsa-miR-320d	4.58	0.19	0.05	5.35	6.08	1.66	33.20

hsa-miR-148a-3p	11.43	8.38	0.12	11.66	13.42	3.39	28.25
hsa-miR-1307-3p	7.87	2.65	0.03	10.43	10.03	0.76	25.33
hsa-miR-99b-5p	5.49	3.3	0.22	7.96	10.36	5.3	24.09
hsa-miR-375	6.51	5.33	0.44	5.05	8.19	8.84	20.09
hsa-miR-151a-3p	10.67	7.58	0.12	14.04	15.28	2.36	19.67
hsa-let-7g-5p	4.86	2.3	0.17	8.5	9.46	1.95	11.47
hsa-miR-3184-3p	8.19	5.85	0.2	12.55	13.27	1.64	8.20
hsa-miR-28-3p	7.18	4.27	0.13	9.72	9.56	0.89	6.85
hsa-miR-222-3p	5.02	3.99	0.49	7.72	9.47	3.34	6.82
hsa-miR-140-3p	3.43	2.41	0.49	8.17	9.86	3.23	6.59
hsa-miR-9-5p	6.59	4.48	0.23	6.36	6.8	1.36	5.91
hsa-let-7i-5p	9.01	6.36	0.16	13.22	12.93	0.82	5.13
hsa-miR-92a-3p	8.13	6.27	0.27	8.91	9.25	1.27	4.70
hsa-miR-378a-3p	6.06	4.22	0.28	8.61	8.42	0.87	3.11
hsa-miR-30d-5p	7.37	6.35	0.49	11.57	12.09	1.44	2.94
hsa-miR-1290	5.09	3.09	0.25	6.08	5.31	0.59	2.36
hsa-miR-146b-5p	5.83	4.51	0.4	9.69	8.7	0.5	1.25
hsa-miR-486-5p	9.24	8.57	0.63	14.39	12.21	0.22	0.35
hsa-let-7f-5p	5.97	6.19	1.17	12.87	11.18	0.31	0.26

Supplementary Figures:

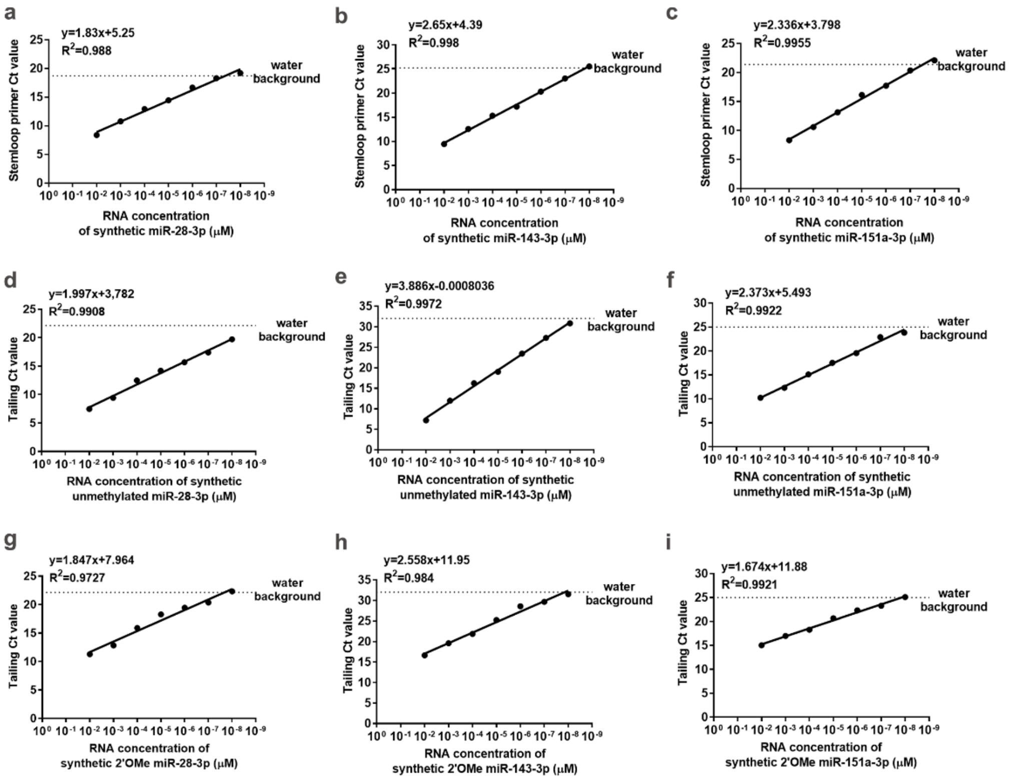

Figure. S1: Amplification delay on 2'OMe miRNAs by poly (A)-tailed qRT-PCR. (a–c) The standard curve of stem-loop primer qRT-PCR for synthetic miR-28-3p (a), miR-143-3p (b), and miR-151a-3p (c); RNA concentration represented the synthetic miRNA concentration before reverse transcription. (d-f) The standard curve of poly (A)-tailed qRT-PCR for detection of unmethylated miR-28-3p (d), synthetic unmethylated miR-143-3p (e), and synthetic unmethylated miR-151a-3p (f); (g–i) The standard curve of poly (A)-tailed qRT-PCR for 2'OMe miR-28-3p (g), 2'OMe miR-143-3p (h), and 2'OMe miR-151a-3p (i). RNA concentration of the miRNA before reverse transcription. The concentration range in the figure is from 10^{-2} to 10^{-8} μM. The upper left corner showed the regression equation and the goodness-of-fit.

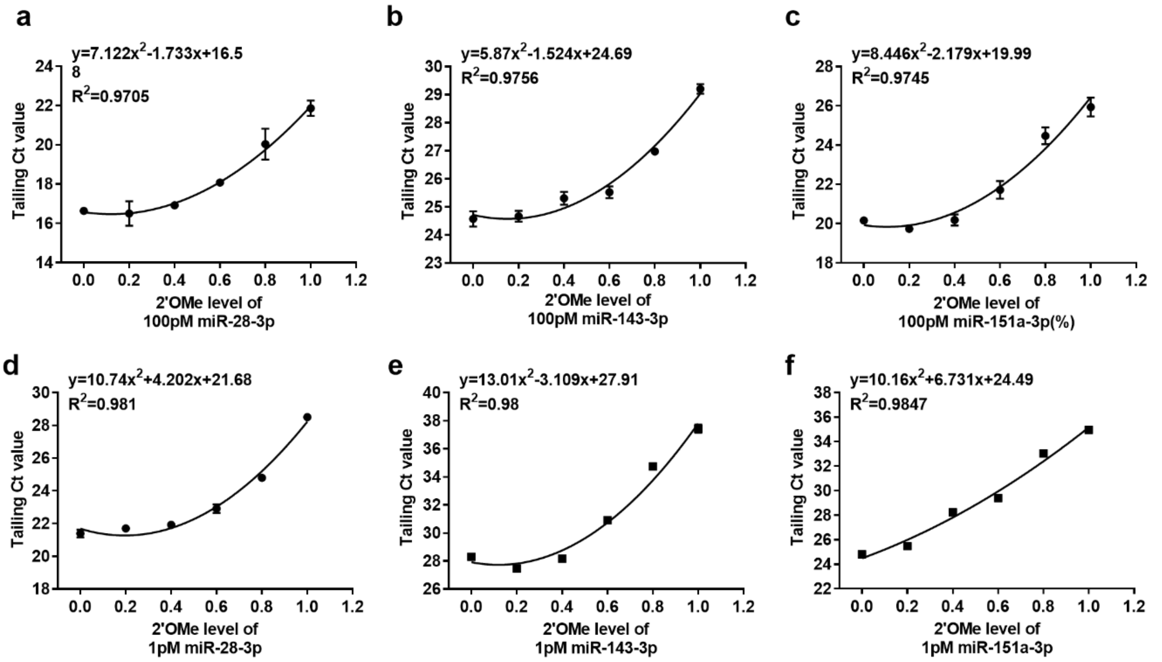

Figure. S2: Ct values of poly (A)-tailed RT-qPCR and methylation ratio fitting of the quadratic curve. Scatter plot of tailing Ct and methylation fraction for synthetic miR-28-3p (a, d), miR-143-3p (b, e), and miR-151a-3p (c, f); The distribution of plots presents a curve; (a-c) Regression curve of Ct value and methylation ratio for different miRNAs at 100 pM; (d–f) Regression curve of Ct value and methylation ratio for different miRNAs at 1 pM. Data were presented as Mean ± SE.

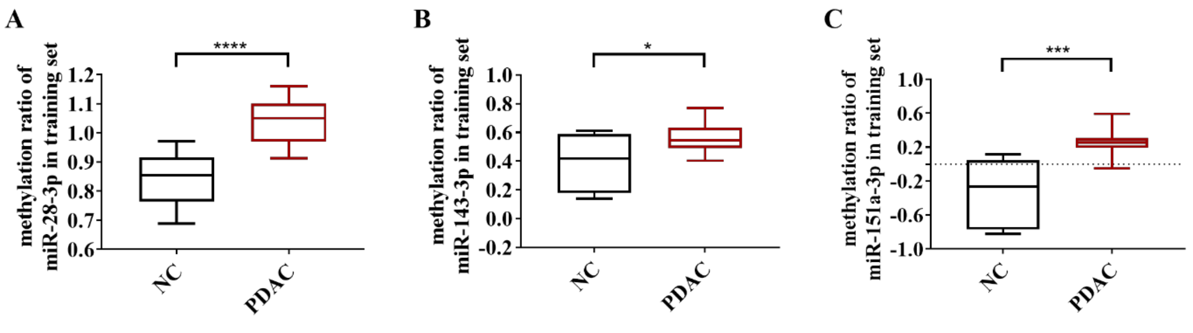

Figure. S3: Methylation ratio of miR-28-3p (a), miR-143-3p (b), and miR-151a-3p (c) in training set. *P < 0.05; ***P < 0.001; ****P < 0.0001.

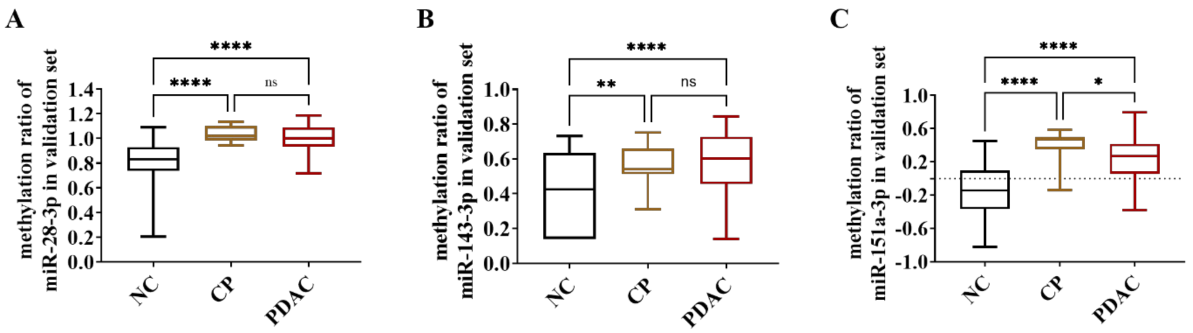

Figure. S4: Methylation ratio of miR-28-3p (a), miR-143-3p (b), and miR-151a-3p (c) in validation set. **P* < 0.05; ****P* < 0.001; *****P* < 0.0001; ns: no significance.
